# Combined platelet-rich plasma and lipofilling treatment provides great improvement in facial skin-induced lesion regeneration for scleroderma patients

**DOI:** 10.1186/s13287-017-0690-3

**Published:** 2017-10-23

**Authors:** Francesco Virzì, Paola Bianca, Alessandro Giammona, Tiziana Apuzzo, Simone Di Franco, Laura Rosa Mangiapane, Maria Luisa Colorito, Dario Catalano, Emanuela Scavo, Annalisa Nicotra, Antonina Benfante, Giuseppe Pistone, Valentina Caputo, Francesco Dieli, Roberto Pirrello, Giorgio Stassi

**Affiliations:** 1 Cellular and Molecular Pathophysiology Laboratory, Department of Surgical Oncological and Stomatological Sciences, University of Palermo, Via del Vespro 131, Palermo, 90127 Italy; 2 Division of Plastic and Reconstructive Surgery Department of Surgical and Oncological Sciences, University of Palermo, Via del Vespro 131, Palermo, 90127 Italy; 3 DiBiMIS, Piazza delle Cliniche, 2, University of Palermo, Palermo, 90127 Italy; 4 Central Laboratory of Advanced Diagnosis & Biomedical Research (CLADIBIOR), University of Palermo, Via del Vespro 131, Palermo, 90127 Italy

**Keywords:** Systemic sclerosis, Platelet-rich plasma, Mesenchymal stem cells, Adipose-derived mesenchymal stem cells, Cell therapy, Lipofilling, Regenerative medicine

## Abstract

**Background:**

The use of stem cells, including mesenchymal stem cells (MSCs), for regenerative medicine is gaining interest for the clinical benefits so far obtained in patients. This study investigates the use of adipose autologous tissue in combination with platelet-rich plasma (PRP) to improve the clinical outcome of patients affected by systemic sclerosis (SSc).

**Methods:**

Adipose-derived mesenchymal stem cells (AD-MSCs) and PRPs were purified from healthy donors and SSc patients. The multilineage differentiation potential of AD-MSCs and their genotypic–phenotypic features were investigated. A cytokine production profile was evaluated on AD-MSCs and PRPs from both healthy subjects and SSc patients. The adipose tissue-derived cell fraction, the so-called stromal vascular fraction (SVF), was coinjected with PRP in the perioral area of SSc patients.

**Results:**

Histopathological and phenotypical analysis of adipose tissue from SSc patients revealed a disorganization of its distinct architecture coupled with an altered cell composition. Although AD-MSCs derived from SSc patients showed high multipotency, they failed to sustain a terminally differentiated progeny. Furthermore, SVFs derived from SSc patients differed from healthy donors in their MSC-like traits coupled with an aberrant cytokine production profile. Finally, the administration of PRP in combination with autologous SVF improved buccal’s rhyme, skin elasticity and vascularization for all of the SSc patients enrolled in this study.

**Conclusions:**

This innovative regenerative therapy could be exploited for the treatment of chronic connective tissue diseases, including SSc.

**Electronic supplementary material:**

The online version of this article (doi:10.1186/s13287-017-0690-3) contains supplementary material, which is available to authorized users.

## Background

Regenerative medicine has been the subject of numerous studies, and to date several successes have been reported in regenerating nonfunctional tissue or damaged organs affected by traumas and congenital and/or acquired disorders [1, 2]. Tissue engineering often resorts to the use of stem cells and/or synthetic scaffold as supporting structures [3, 4].

Mesenchymal stem cells (MSCs), thanks to their multilineage differentiation potential, are the most promising candidates for regenerative medicine techniques [5, 6]. Recent findings have shown that adipose tissue is an important source of MSCs [7]. Therefore, this prompted great interest in the scientific community, leading to the discovery of advanced techniques used for the collection and isolation of MSCs from lipoaspirates [8, 9], and their use in the clinic. Lipofilling is a surgical protocol that was standardized by Dr Sidney Coleman in 1997 [10], aiming at the transfer of autologous adipose tissue. The current lipofilling technique consists of three phases: subcutaneous tumescent liposuction from the abdomen, medial knee, or trochanter regions; centrifugation of the lipoaspirate sample to remove blood elements and the oil fraction from adipose components; and autologous injection of “purified” adipose tissue [11, 12]_._

Adipose tissue is composed of mature adipocytes, fibroblasts, adipose-derived mesenchymal stem cells (AD-MSCs), immune system cells, and endothelial cells, which are grouped as the stromal vascular fraction (SVF) [13]. The presence of all these cellular elements, in particular the large number of AD-MSCs, makes the SVF the most prominent candidate for lipofilling therapeutic success [8, 14–16].

In fact, AD-MSCs secrete high levels of growth factors and cytokines such as vascular endothelial growth factor (VEGF) and hepatocyte growth factor (HGF), which are all crucial molecules for lipotransfer engraftment and tissue regeneration [3–5, 17].

AD-MSCs are endowed with great multilineage differentiation potential and relevant regenerative properties [18–20]. AD-MSCs are able to grow in suspension as spheroids without serum and they can be identified through a high expression level of the CD271 marker [21, 22].

Systemic sclerosis (SSc) is a chronic connective tissue disease associated with autoimmune pathogenesis and unknown etiology [23, 24]_._ It causes a widespread microangiopathic vascular dysfunction and morphofunctional alterations [25].

The most affected districts in SSc patients are the joints of distal limbs and perioral and malar areas. A gradual reduction was observed in the opening and extension rates of the labialis rhyme, due to fibrosis and loss of endothelium integrity, inflammatory mononuclear infiltrate, and high production of reactive oxygen species (ROS) [25–27].

These conditions promote a compensatory VEGF overproduction by the endothelium. In healthy subjects the increase of VEGF is coupled with platelet-derived growth factor (PDGF), endothelin-1 (ET-1), transforming growth factor beta (TGF-β), connective tissue growth factor (CTGF), and monocyte chemoattractant protein-1 (MCP-1) production, thus promoting angiogenesis. SSc patients show a high production of VEGF, which is not however followed by an improvement of endothelial capillarity, giving rise to telangiectasia [28, 29]. Being that a single lipofilling treatment usually is not sufficient in order to obtain efficient regeneration in SSc patients, a combination of autologous tissue together with an abundant cytokine source would be preferable [30–33].

Platelet-rich plasma (PRP) consists of a gel fraction obtained from peripheral blood. PRP contains a high number of platelets, cytokines, and growth factors. Different studies have shown that PRP promotes coagulation and wound healing, exerting an antiphlogistic effect on the acceptor site and hence stimulating rapid tissue regeneration [34, 35]. Several studies have demonstrated that PRP has a beneficial impact on the regenerative potential of MSCs, and that the combined use of PRPs and lipoaspirates increases graft survival while maintaining the plumping effect in breast reconstruction [36, 37].

However, no study has yet investigated the synergistic effect of lipoaspirate and PRP injections on SSc patients. There has been no in-depth study performed into their effect at a molecular level, in particular with regard to the differences between SVFs and PRPs in healthy individuals as opposed to SSc patients.

Our study is the first to assess the positive effects of the combined use of autologous lipoaspirate and PRP, in treatment of typical perioral and malar alterations in SSc patients. Here we compare the multilineage differentiation potential of AD-MSCs from healthy subjects and SSc patients. Moreover, we characterized PRPs and SVFs derived from healthy subjects as well as SSc patients in order to predict their possible contribution in tissue regeneration.

Finally, we tested the positive effect of combined SVF–PRP treatment on SSc patients. SSc patients were monitored for 3 months with periodical evaluations. All clinical outcome evaluations have highlighted a widespread and progressive improvement in morphological and functional malar and perioral alterations. Our results suggest that this combined treatment could be considered promising for SSc patients.

## Methods

### Patient selection

Patients were treated in compliance with our department’s policy, following patient’s written consent on adipose tissue harvest and its use for research purposes. The study was approved by the ethics committee Palermo-1 Polyclinic Paolo Giaccone of Palermo with report N°1/2016.

A group of six SSc patients affected by cutaneous systemic sclerosis (dcSSc) were selected. These subjects are between the ages of 41 and 63 without malignancies, not pregnant, not lactating or making use of immune modulator drugs, and not having undergone antiplatelet and/or vasodilator treatments in the last 20 days (Table 1). For our healthy control group, we selected five lipoaspirate samples from 50 of our healthy subjects (16 males, 34 females) [20] (Table 1). The selection of healthy samples was based on their capability to give rise to AD-MSCs.

**Table 1:** Case description

Case	Gender	Liposuction area	Age/disease duration^a^ (years)	Lipoaspirate (ml)	Blood (ml)	Platelet-rich plasma (ml)
Healthy subjects						
#2	Male	Medial knee	85	110	20	10
#11	Male	Abdomen	43	120	20	12
#12	Female	Medial knee	50	92	25	9
#14	Female	Abdomen	54	93	20	10
#19	Female	Abdomen	44	120	25	10
Sclerodermic patients						
#1	Female	Medial knee	6	110	25	12
#2	Female	Medial knee	16	140	25	12
#3	Female	Medial knee	8	92	25	12
#4	Male	Abdomen	3	93	20	9
#5	Male	Abdomen	3	120	25	9
#6	Female	Medial knee	20	90	25	13

^a^Age of healthy subjects; disease duration for sclerodermic patients

### Adipose tissue and culture

The withdrawal of adipose tissue was performed under conscious sedation through local infiltration of 150 cm^3^ of Klein solution. A 10-gauge cannula connected to a 10-ml syringe with luer-lock-type attack was used for the liposuction. Then 90–140 cm^3^ of subcutaneous adipose tissue was extracted from each patient. Lipoaspirates from healthy subjects were obtained using a liposuction procedure. These subjects had been previously screened to ensure the absence of chronic illness. The sample was centrifuged for 5 min at 2700 rpm. After centrifugation, the samples were stratified into three layers, the upper layer representing the oily component, the middle one consisting of a solid tissue layer, and the bottom layer composed of MSCs and blood elements. Lipoaspirate samples deprived of the blood and oily phases were divided into two fractions: 12–40 cm^3^ were used for in-vitro analyses and 22–50 cm^3^ for the surgical procedure.

Lipoaspirates from SSc patients and healthy donors were digested for 30 min at 37 °C, in the presence of collagenase (1.5 mg/ml; GIBCO) and hyaluronidase (20 mg/ml; Sigma). The digested samples were centrifuged and washed with PBS. The obtained pellet was suspended in serum-free stem cell-specific medium, supplemented with bFGF (10 ng/ml; Sigma) and EGF (20 ng/ml; Sigma), in ultra-low adhesion culture flasks (Corning) as described previously [38]. In these conditions, cells grew as floating spheroids, called AD-MSCs [20]. StemPro™ Human Adipose-Derived Stem Cells (ADSCs; ThermoFisher), cultured as the manufacturer recommended, were used as the MSC control.

### Flow cytometry

AD-MSCs and freshly purified samples were harvested, washed twice in PBS, and then stained with conjugated antibodies against CD271-ALEXA FLUOR 647 (C40-1457, IgG1k; BD), CD44-FITC (G44-26, IgG2bk; BD), CD90-PE (5E10, IgG1k; BD), and CD45-FITC (5B1, IgG2a; Miltenyi), or with purified primary antibodies against CD29 (MAR4, IgG1k; BD), CD9 (M-L13, IgG1k; BD), and CD73 (AD2, IgG1k; BD). Where necessary, cells were then labeled with goat anti-mouse IgG-Alexa Fluor 488 secondary antibody (Invitrogen). Corresponding isotype-matched antibodies, CD3-Alexa Fluor 647 (UCHT1, IgG1k; BD), CD3-FITC (UCHT1, IgG1k; BD), CD3-PE (UCHT1, IgG1k; BD), CD8 (BW135/80, IgG2a; Miltenyi), and Purified CD3 (UCHT1, IgG1k; BD), were used as negative controls. Samples were acquired using an AccuriC6 (BD) flow cytometer. All data were analyzed by FlowJo software (Tree-Star).

### Tissue morphological analysis

Undigested lipoaspirates collected from SSc patients and healthy subjects were included in paraffin-embedded sections and then stained for hematoxylin and eosin (H&E) according to standard protocols.

### Osteogenic, chondrogenic, and adipogenenic differentiation

SSc and healthy AD-MSCs were plated into 24-well cell culture plates (50 × 10^3^ cells/well). Cells were allowed to adhere and cultured in the presence of STEMPRO® Osteogenesis, Chondrogenesis, or Adipogenesis Differentiation media (Invitrogen) for up to 28 days. Cell viability, adhesion, and differentiation capacity were determined by daily observation using optical microscopy. After 28 days the samples were fixed in 2% PFA for 30 min at 37 °C and washed in PBS. The osteogenic differentiation was assessed by performing von Kossa staining for calcium deposition (Polysciences). Chondrogenic differentiation was assessed through alcian blue staining. Chondrocytes and osteoblasts were then counterstained with nuclear fast red (Polyscience). The adipogenic phenotype was assessed via AdipoRed assay (Lonza) for 10 min at RT, nuclei were counterstained with DAPI (Thermofisher), and the staining was observed using a fluorescence microscope (Olympus BX 60).

### Gene expression

Total RNA of SSc and healthy AD-MSCs was extracted using the RNeasy Mini Kit (Qiagen) and 0.8 μg of each sample was retro-transcribed into cDNA using the RT^2^ First strand kit (Qiagen) as recommended by the manufacturer. Expression analysis of MSC genes was performed through RT2 profiler PCR array (Qiagen PAHS-082Z) according to the manufacturer’s instructions. Data analysis was performed by RT^2^ Profiler PCR Array Data Analysis version 3.5 (Qiagen).

### Activated PRP preparation

Venous blood samples (25 cm^3^) were collected from healthy and SSc donors. PRP samples were isolated using Arthrex ACP syringes. These syringes were previously filled with 1.5 cm^3^ of 3.8% sodium citrate to prevent clotting of sample. Blood samples were processed with Rotofix 32-A (Hettich) according to the manufacturer’s instructions. Approximately 10–12 cm^3^ of PRP was obtained.

### SVF and PRP cytokine analysis

SVFs were collected from the upper phase of lipoaspirates following digestion. PRPs were harvested from blood samples. PRP and SVF samples were filtered using 0.45-μm filters (Corning) and then stored at –80 °C until use. After thawing, all the samples were analyzed in a single run in which cytokine quantification was assessed using the Bio-Plex Pro™ Human Cytokine 21-plex and 27-plex Assay (Bio-Rad). Raw data were analyzed by Bio-Plex Software (Bio-Rad).

### Surgical techniques

The PRP was injected in perioral and malar areas, divided proportionally between the right and left sections of the face. After 10 min, in the same PRP injection spot, the lipo-transfer was performed with a 15-gauge infiltration cannula.

The injection was performed slowly to limit mechanical damage, and then 500 mg of Solu-Medrol was administered to minimize tissue inflammation and postoperative edema. Compressive medication was applied to the area of injection. Patients remained under observation for 24 hours. In both the abdomen and medial knee sampling sites, a stitch was applied with Ethilon 4-0 thread. The medial knee point was covered by elastocompressive stockings for about 4 weeks to reduce edema and facilitate tissue stranding, while the abdomen was protected by an abdominal sheath.

### Instrumental analysis

Patients were evaluated preoperatively and postoperatively for a period of 3 months. Morpho-dynamic analysis was performed through the acquisition of digital images and with electronic caliber (Profi Line), checking the degree of opening and extension of the labial rhyme. Skin stretching capacity was measured using the Elastometer-EM 25 (G.F. Secchi), and was calculated using the formula:

$$ \mathrm{Elasticity}=\left(\left(b-a\right)/a\right)\times 100, $$

where *a* is the maximum depth of penetration of the skin and *b* is the relaxation of the skin. Videodermatoscopic analysis for cutaneous vascularization was conducted using a videodermatoscope (Easyscan model; Pico).

### Clinical outcome evaluation

The postoperative evaluation consisted of two phases. A t the initial inspection (first week), the face bandages were removed, the stitches were checked and then removed from the sampling areas, the site was disinfected, and gauze with gentamicin was applied. The compression stockings and abdominal sheath were worn for 1 month. During the second check (after 3 months), the opening and extension indexes of the labial rhyme were evaluated by caliper. The cutaneous elasticity index of the upper left lip and of the left upper cheek was evaluated using a skin suction elastometer. To document the change in vascularization, two snapshots were taken of the upper left lip through the videodermatoscope both in artificial and UV light. Patient’s satisfaction was rated through a questionnaire composed of 14 points, in which the numerical evaluation scale used was “1” being the lowest and “10” being the highest level of satisfaction.

### Statistical analysis

Data are expressed as mean ± standard deviation (SD). Statistical significance was calculated by applying Student’s *t* test. Significance levels were indicated as *p* values.

## Results

### SSc adipose tissue is characterized by a different architecture and cell composition

**Fig. 1:**
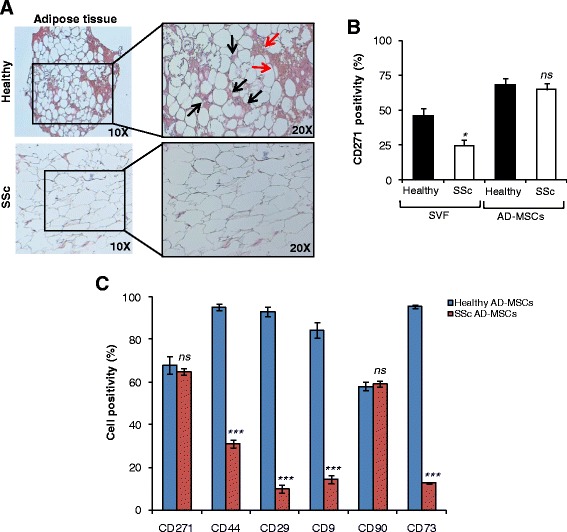
SSc adipose samples show peculiar features and different tissue morphology. **a** Representative H&E staining on paraffin-embedded sections of freshly isolated adipose tissue from healthy donors and SSc patients. Arrows indicate MSCs (black) and blood vessels (red). **b** CD271 positivity in freshly isolated SVF and long-term propagated AD-MSCs from healthy donors and SSc patients, performed by flow cytometry. Data are mean ± SD of three independent experiments. **c** Flow cytometry analysis of cell positivity percentage for CD271, CD44, CD29, CD9, CD90, and CD73, performed on AD-MSCs of healthy subjects (blue) and SSc patients (red). Percentage values in **b** and **c** represent the percentage of parent population. Bars represent mean ± SD of three independent experiments. *p < 0.05, ***p < 0.001, ns nonsignificant. AD-MSC adipose-derived mesenchymal stem cell, SSc systemic sclerosis, SVF stromal vascular fraction

In order to define whether adipose tissue from SSc patients differs from that present in the healthy subjects, we analyzed the tissue at the histopathological level on paraffin-embedded sections. Of note, we observed a clear disorganization of cell structure in the adipose tissue of all SSc patient samples analyzed, as compared to those of healthy donors (Fig. 1a).

While the SVF from SSc patients showed significantly lower expression levels of the putative MSC marker CD271 [20] compared to that isolated from healthy subjects, this variation was not evident in the long-term propagated AD-MSCs, which were grown in vitro as spheroids (Fig. 1b). These findings suggest that mesenchymal stem-like cells expressing CD271 are enriched during selective culture conditions.

Isolated and in-vitro propagated AD-MSCs from adipose tissue of SSc patients expressed CD44, CD29, CD9, and CD73 to a lesser extent than those derived from healthy individuals (Fig. 1c).

These data suggest that SSc patients suffer from a disorganized adipose tissue structure, characterized by a smaller number of cells with a stem-like phenotype. For these reasons, a detailed and thorough analysis of the multipotential capacity of SSc AD-MSCs is strongly required.

### SSc AD-MSCs are multipotent but unable to generate mature functional phenotypes

**Fig. 2:**
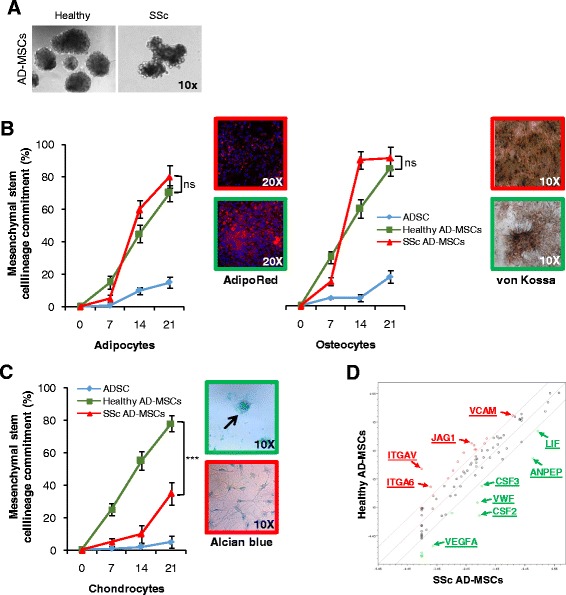
SSc AD-MSCs display great multilineage differentiation ability but an incomplete mesenchymal maturation phenotype. **a** Representative phase-contrast microscopic analysis of AD-MSCs from healthy subjects and SSc patients. **b** Percentage of adipocyte and osteocyte differentiation of AD-MSCs derived from healthy donors and SSc patients (ADSC-derived mature cells used as control). Right panels show morphological analysis of the AD-MSC adipocyte (AdipoRed staining, 20× magnification) and osteocyte (von Kossa staining, 10× magnification) differentiated progeny from healthy subjects (green) and SSc patients (red). **c** Percentage of chondrocyte differentiation of AD-MSCs derived from healthy donors and SSc patients as in **b**. Right panel shows morphological analysis of the AD-MSC chondrocyte (alcian blue staining, 10× magnification) differentiated progeny from healthy subjects (green) and SSc patients (red). Arrow indicates the chondrocyte agglomerates. **d** Scatter plot of significantly differentially expressed mesenchymal-related genes in AD-MSCs from healthy donors and SSc patients. Data are mean ± SD of three independent experiments. ***p < 0.001, ns non significant. AD-MSC adipose-derived mesenchymal stem cell, ADSC STEMPRO® Human Adipose-Derived Stem Cells, SSc systemic sclerosis

To investigate the multilineage differentiation capabilities of AD-MSCs, we allowed them to differentiate toward adipocytes, osteocytes, and chondrocytes in the presence of a specific differentiation tissue culture medium (Fig. 2a). Following specific differentiation, although AD-MSCs from SSc patients showed a great percentage of cell lineage commitment, they displayed an aberrant multipotent differentiation capacity in adipocytes and osteocytes when compared with AD-MSCs from healthy subjects (Fig. 2b). This aberrancy consisted of an incomplete maturation of the adipo-progenitor and osteo-progenitor cells as highlighted by the presence of small lipid droplets (AdipoRed staining) and the absence of calcium deposits (von Kossa staining), respectively (Fig. 2b). Alcian blue analysis revealed that the functional differentiation of chondrocytes was impaired in the adipose spheroid-derived adherent cells of SSc patients. This was shown by a significant reduction in cell commitment coupled with a lack of chondrocyte agglomeration (Fig. 2c). In accordance with the functional alterations observed in AD-MSCs from SSc patients, a downregulation of MSC-related genes underlined a blockage in a late stage of commitment, impeding the completion of the maturation process (Fig. 2d).

In particular, we found a downregulation of the master regulator of cell differentiation JAG1, a Notch ligand, as well as an upregulation of proinflammatory factors such as CSF2 (GM-CSF) and CSF3 (G-CSF) in the AD-MSCs of SSc patients. Thus, these data suggest that the inflammatory state, which characterizes SSc patients, could cause an impairment of the physiological differentiation mechanisms.

### SSc samples have a high content of proinflammatory cytokines and a shortage of angiogenic factors

**Fig. 3:**
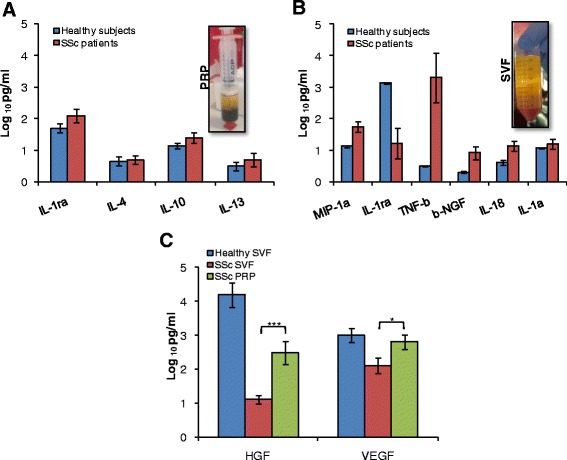
SVF and PRP of SSc patients and healthy subjects show different paracrine factors. **a** Growth factors and cytokines released by undigested PRP (right panel) collected from healthy donors and SSc patients. Bars represent mean ± SD of three independent experiments performed on three different samples from both healthy subjects and SSc patients. **b** Analysis of growth factor and cytokine levels in undigested SVF (right panel) collected as in **a**. Bars represent mean ± SD of three independent experiments performed on four different samples from both healthy subjects and SSc patients. **c** Analysis of HGF and VEGF levels in the SVF from healthy subjects and SSc patients, and PRP from SSc patients. Data are mean ± SD of three independent experiments performed using four different biological samples from both healthy subjects and SSc patients. *p < 0.05, ***p < 0.001. AD-MSC adipose-derived mesenchymal stem cell, HGF hepatocyte growth factor, PRP platelet-rich plasma, SSc systemic sclerosis, SVF stromal vascular fraction, VEGF vascular endothelial growth factor

Having a high regard for the proinflammatory phenotype shown by the AD-MSCs purified and propagated from SSc patients, we sought to investigate the microenvironmental cytokines produced by platelets (PRP) and cells within SVF. Importantly, this characterization was performed prior to treating patients with Solumederol. Higher levels of MIP-1a, TNF-β, b-NGF, IL-18, and IL-1α proinflammatory cytokines and lower levels of IL-1ra anti-inflammatory cytokine were found in the SVF isolated from SSc patients when compared with the corresponding fraction from healthy subjects (Fig. 3a). Analysis of cytokines present in PRPs from four healthy donors and four SSc patients showed comparable expression levels of anti-inflammatory cytokines, including IL-13, IL-10, IL-4, and IL-1ra (Fig. 3b). Although VEGF and HGF were barely produced by the SVF of SSc patients, their presence was significantly higher in the related PRP (Fig. 3c).

These data reveal that cytokines released by cells present in the SVF of SSc patients are likely to boost the inflammation process that is responsible for the failure of autologous SVF injection alone as regenerative treatment for SSc patients. Consistently, the autologous administration of SVF in combination with PRP could lead to the inhibition of the inflammation favoring the adipose tissue regeneration process.

### SSc patients treated with PRP and SVF showed a substantial increase of morphofunctional parameters and capillary density in the maxillofacial region

**Fig. 4:**
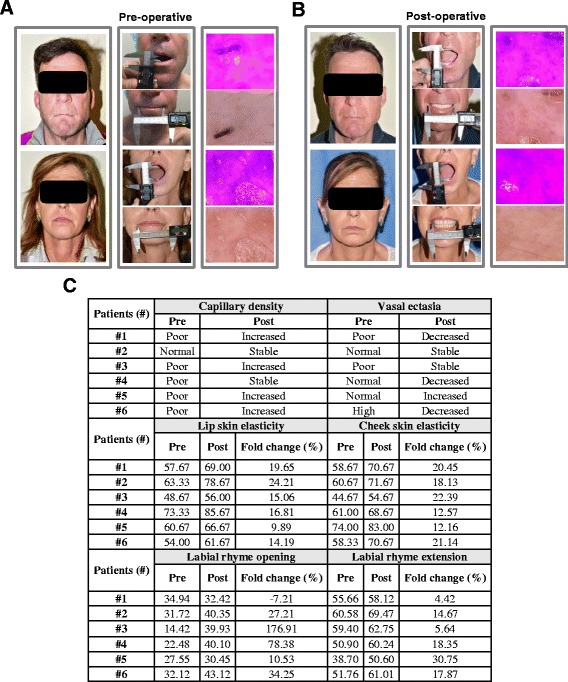
Combinatorial treatment benefits for SSc patients. **a** SSc patient preoperative controls (upper Patient #3, lower Patient #6). Malar and perioral area morphological evaluation (left panels). Evaluation of open and extension rates of the labial rhyme performed with an electronic gauge (middle panels). Left margin lip capillary density and vascular ectasia evaluation (right panels). **b** SSc patient postoperative controls as reported in **a**. **c** Videodermatoscopic surgery: capillary density values (upper left) and vascular ectasia parameters (upper right). Clinical elastomeric indexes: perioral area percentage increase (middle left) and malar area increase percentage (middle right). Operative labial rhyme opening and extension rates: average percentage of the variation openings parameter (down left) and extension parameter (down right)

Six SSc patients were enrolled to test the efficacy of the combinatorial administration of autologous SVF and PRP in seeking improvement in morphological and functional aspects of the facial area (Table 1). Notably, perioral and malar areas coinjected with autologous SVF and PRP led to a substantial increase in skin elasticity (16.64% for the lip and the 17.80% for the cheek), resulting in a marked improvement of the opening and extension benchmarks of the labial rhyme (Fig. 4a, b left and middle panels, c).

The enhancement of morphological facial parameters was maintained up to 3 months after the autologous administration of SVF in combination with PRP (Fig. 4c). Longitudinal skin wrinkles of the upper lip were ironed out and the profile of both lips was more harmonious, less tense, full, and softer. The evaluation of the labial rhyme opening rate showed an increase in five of the six enrolled patients, while six of six showed a labial rhyme extension increment (Fig. 4c). In accordance with our ex-vivo findings, the videodermatoscope vascular examination of SSc patients who had undergone the combinatorial treatment showed an increase of capillary density (4/6 patients) and a decreased vascular ectasia (2/6 patients), thus suggesting an induction of the neoangiogenesis processes (Fig. 4a, b, right panels). The perception of patient care was promising for all of the analyzed parameters (Tables 2 and 3).

**Table 2:** Perception of patient care

Mr/Mrs...............................................	Date.............................
Please express yours opinions with a number from 1 to 10, where 1 corresponds to dissatisfied and 10 very satisfied. 1. How much were you satisfied by the aspect of your lips (before surgery)? _____ 2. How much were you satisfied by the skin elasticity of the perioral area (before the intervention)? _____ 3. How much were you satisfied by the aspect of the malar area (before surgery)? _____ 4. How much were you satisfied by the skin elasticity of the malar area (before surgery)? _____ 5. Indicate the discomfort felt during the opening of the labial rima (before surgery). _____ 6. Indicate the discomfort felt during the extension of the labial rima (before surgery). _____ Rating 3 months. Date……………………….. 1. How much are you satisfied by the aspect of your lips (after surgery)? _____ 2. How much are you satisfied by the skin elasticity of the perioral region (after surgery)? _____ 3. How much are you satisfied by the aspect of the malar (after surgery)? _____ 4. How much are you satisfied by the skin elasticity of the malar region (after surgery)? _____ 5. Indicate the discomfort you feel during the opening of the labial rhyme (after surgery). _____ 6. Indicate the discomfort you feel during the extension of the labial rhyme (after surgery). _____ General evaluation. 7. Will you do again this type of procedure? _____ 8. Would you recommend this procedure to other people with the same disease? _____

**Table 3:** Survey satisfaction of patients: preoperative and postoperative satisfaction values

Parameter	Preoperative index mean (1–10)	Postoperative index mean (1–10)
Appearance of the lips	3	8
Perioral skin elasticity	3.16	8.83
Malar area aspect	3.33	9.33
Malar skin elasticity	3.16	9.5
Labial rhyme opening discomfort	2.83	7.83
Labial rhyme extension discomfort	3	8

These data show that a single shot of coinjected autologous SVF and PRP in the facial area of SSc patients could be considered a promising regenerative therapy.

## Discussion

Despite the high number of MSCs in adipose tissue and the many documented therapeutic successes in the fields of tissue engineering, the exclusive use of lipofilling has shown an inefficient regeneration in SSc patients [35]. Here, we demonstrate that the injection of autologous adipose tissue-derived SVF, enriched in MSCs, in combination with PRP, into the perioral and malar areas of SSc patients not only improved the facial morphofunctional issues, but significantly enhanced the buccal’s rhyme, skin elasticity, and vascularization.

The use of MSCs has been introduced relatively recently in the clinical practice of regenerative medicine. Several studies highlighted their self-renewal, multilineage differentiation capacity, and immunomodulatory properties [39]. As well as being multipotent stem cells, the MSCs are able to differentiate into different cell types including adipocyte, chondrocyte, osteoblast, and neuron-like cells [39]. Among their properties, their accessibility and easy expansion suggest that use of MSCs may be a useful therapeutic approach for several disorders.

Nowadays adipose tissue is considered an innovative source of MSCs suitable for cell-based therapy. Autologous micrografting of AD-MSCs was recently demonstrated to induce positive effects on SSc patients [40]. Griffin et al. [41] recently established that AD-MSCs from healthy individuals and SSc subjects present the same phenotype and differentiation capacity, while migration and proliferation are impaired.

Notably, significant difference in the SVF composition characterized the adipose tissue obtained from SSc patients as compared to that from healthy subjects. The adipose compartment of SSc patients showed a disruption of its peculiar morphology and a bare presence of mesenchymal cells, thus suggesting that the inflammatory microenvironment, typical of this systemic disease, could affect the architecture and the adipose cell reservoir. Indeed, the abnormal presence of proinflammatory cytokines in the adipose tissue compartment of SSc patients impaired the differentiation and maturation of MSCs toward the adipose phenotype. Accordingly, mesenchymal stem cell-like traits of MSCs purified from SSc patients significantly differed from those of healthy subjects.

Although both AD-MSCs derived from healthy and SSc SVFs contained a subpopulation positive for CD271, a putative adipose stem cell marker [20], the SSc AD-MSCs lacked the ability to differentiate into functional mesenchymal cellular types. Cells within the SVF in SSc patients, according to their MSC-related gene profile and proliferation rate, are likely to be in a late stage of commitment, which impairs the ultimate phase of cell differentiation. Several transcription factors are known to play a crucial role in the last steps of differentiation, thus regulating cell maturation of AD-MSCs in adipocytes, osteocytes, or chondrocytes, such as CCAAT/enhancer-binding protein alpha (CEBPα) [42], runt-related transcription factor 2 (RUNX2) [43], or SOX-9 [44], respectively. Likewise, the failure of AD-MSCs from SSc patients in functional differentiation could also depend on a delayed maturation block due to a dysregulation at the transcriptional level.

Vascular damage and alteration of subcutaneous microcirculation caused by angiogenic factor deficiency are described to be among the major clinical signs of SSc patients [10, 32]. The SSc SVF compartment defected in the production of several master regulators of angiogenesis, which is crucial for both engraftment and tissue regeneration. Despite the significant advances in the therapeutic options for the treatment of SSc patients, the novel pharmacological compounds that target the hypoxia signaling pathways and immune response have not yet proven beneficial to quality of life.

## Conclusions

Our evidence supports the hypothesis that coinjection of autologous SVF and PRP in SSc patients could provide the correct balance of angiogenic and growth factors to improve tissue regeneration, thus representing an optimal combinatorial therapy against SSc.

## Additional files

Additional file 1: Figure S1. showing AD-MSCs from both healthy subjects and SSc patients with increased levels of CD271 expression. Representative dot plot showing the expression of CD271 corresponding isotype match control in freshly isolated SVF and long-term propagated AD-MSCs from healthy subjects (upper panels) and SSc patients (lower panels), performed by flow cytometry. (PDF 205 kb)

Additional file 2: Figure S2. showing AD-MSCs from SSc patients with decreased levels of mesenchymal stem cell markers. Representative dot plot showing the expression of CD44, CD90, CD271, CD29, CD73, and CD9 in AD-MSCs from healthy subjects (upper panels) and SSc patients (lower panels). Small boxes show isotype match control staining. (PDF 316 kb)

## Abbreviations

AD-MSC: Adipose-derived mesenchymal stem cell
CEBPα: CCAAT/enhancer-binding protein alpha
CTGF: Connective tissue growth factor
ET-1: Endothelin-1
H&E: Hematoxylin and eosin
HGF: Hepatocyte growth factor
MCP-1: Monocyte chemoattractant protein-1
MSC: Mesenchymal stem cell
PDGF: Platelet-derived growth factor
PRP: Platelet-rich plasma
ROS: Reactive oxygen species
RUNX2: Runt-related transcription factor 2
SSc: Systemic sclerosis
SVF: Stromal vascular fraction
TGF-β: Transforming growth factor beta
VEGF: Vascular endothelial growth factor
