# Supplementary figures and images for: Combined platelet-rich plasma and lipofilling treatment provides great improvement in facial skin-induced lesion regeneration for scleroderma patients
Source: Stem Cell Res Ther. 2017 Oct 23;8:236. doi: 10.1186/s13287-017-0690-3 (PMC5651639; doi:10.1186/s13287-017-0690-3)

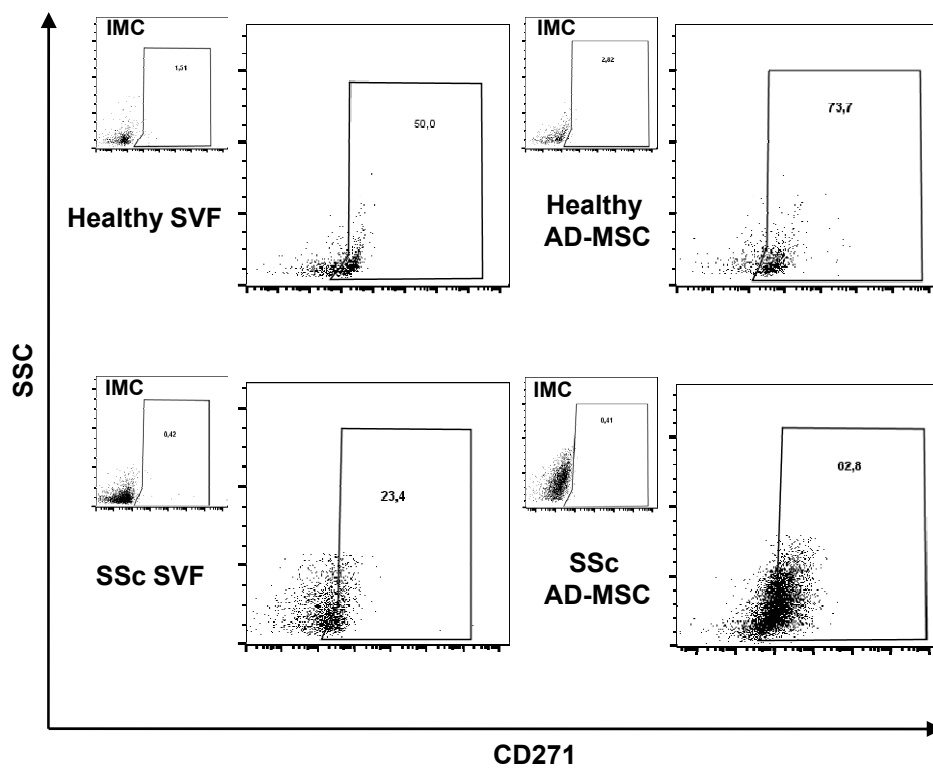

**Figure S1**

Supplement: Supplementary file 1 — showing AD-MSCs from both healthy subjects and SSc patients with increased levels of CD271 expression. Representative dot plot showing the expression of CD271 corresponding isotype match control in freshly isolated SVF and long-term propagated AD-MSCs from healthy subjects (upper panels) and SSc patients (lower panels), performed by flow cytometry. (PDF 205 kb) [file 13287_2017_690_MOESM1_ESM.pdf]

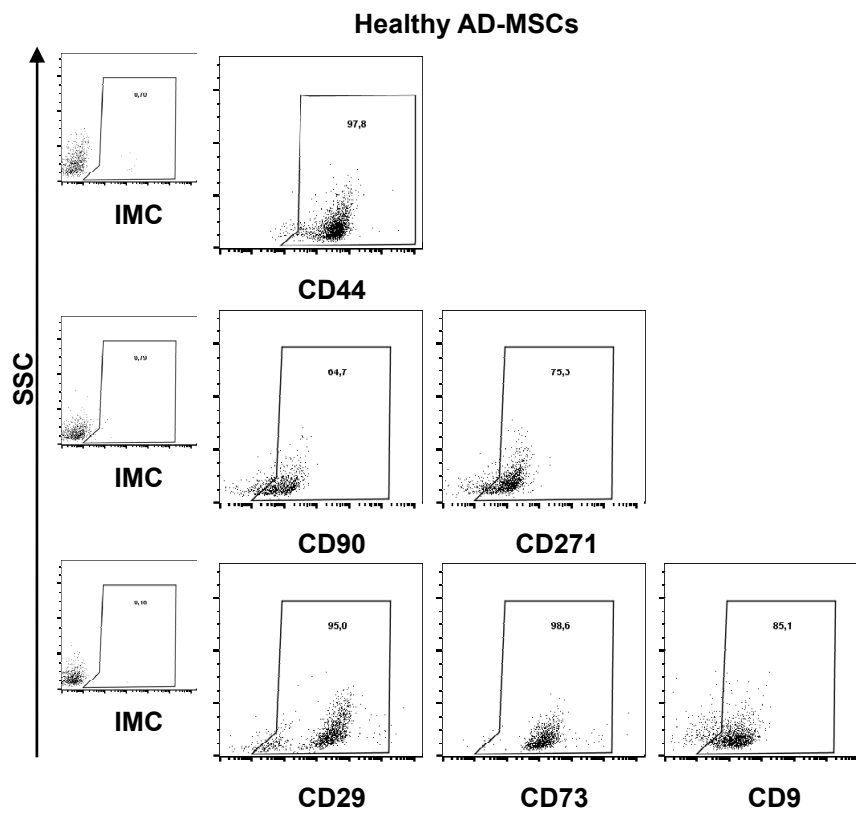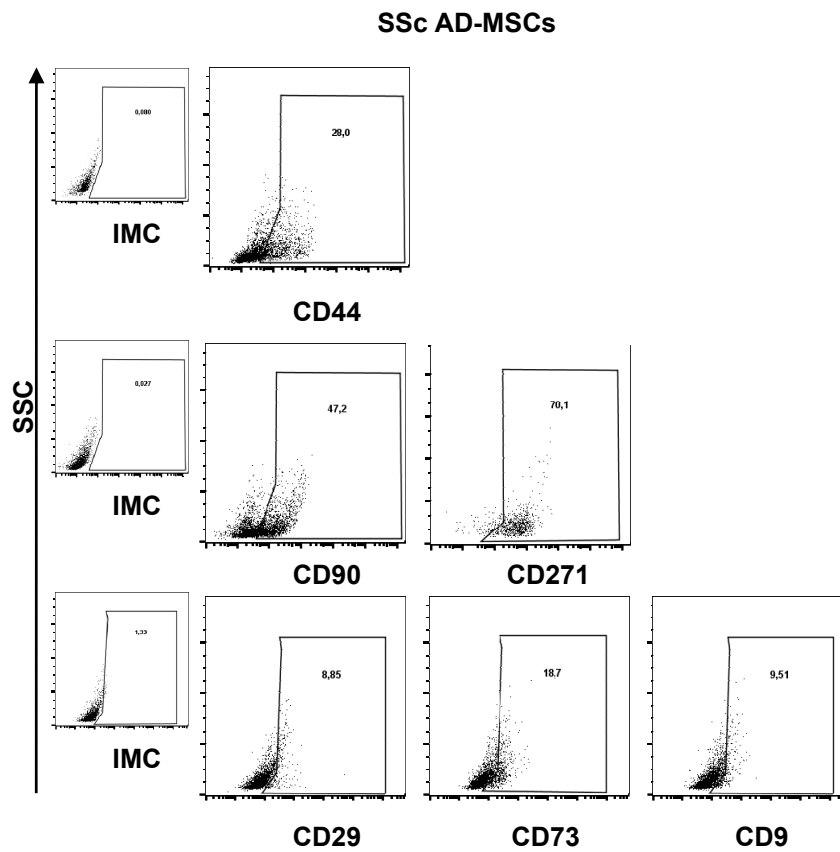

**Figure S2**

Supplement: Supplementary file 2 — showing AD-MSCs from SSc patients with decreased levels of mesenchymal stem cell markers. Representative dot plot showing the expression of CD44, CD90, CD271, CD29, CD73, and CD9 in AD-MSCs from healthy subjects (upper panels) and SSc patients (lower panels). Small boxes show isotype match control staining. (PDF 316 kb) [file 13287_2017_690_MOESM2_ESM.pdf]
